# PoseNet++: A multi-scale and optimized feature extraction network for high-precision human pose estimation

**DOI:** 10.1371/journal.pone.0326232

**Published:** 2025-06-25

**Authors:** Chao Lv, Geyao Ma

**Affiliations:** College of Electronic Information Engineering, Changchun University of Science and Technology, Changchun, China; Guangdong University of Petrochemical Technology, CHINA

## Abstract

Human pose estimation (HPE) has made significant progress with deep learning; however, it still faces challenges in handling occlusions, complex poses, and complex multi-person scenarios. To address these issues, we propose PoseNet++, a novel approach based on a 3-stacked hourglass architecture, incorporating three key innovations: the multi-scale spatial pyramid attention hourglass module (MSPAHM), coordinate-channel prior convolutional attention (C-CPCA), and the PinSK Bottleneck Residual Module (PBRM). MSPAHM enhances long-range channel dependencies, enabling the model to better capture structural relationships between limb joints, particularly under occlusion. C-CPCA combines coordinate attention (CA) and channel prior convolutional attention (CPCA) to prioritize keypoints’ regions and reduce the confusion in complex multi-person scenarios. The PBRM improves pose estimation accuracy by optimizing the receptive field and convolutional kernel selection, thus enhancing the network’s feature extraction capabilities in multi-scale and complex poses. On the MPII validation set, PoseNet++ improves the PCKh score by 3.3% relative to the baseline 3-stacked hourglass network, while reducing the number of model parameters and the number of floating-point operations by 60.3% and 53.1%, respectively. Compared with other mainstream human pose estimation models in recent years, PoseNet++ achieves the state-of-the-art performance on the MPII, LSP, COCO and CrowdPose datasets. At the same time, the model complexity of PoseNet++ is much lower than that of methods with similar accuracy.

## 1. Introduction

Human pose estimation (HPE) is a very popular direction recently. Specifically, HPE serves as the foundation for many directions in the field of human pose, such as action recognition [1,2], human-computer interaction, and cross-domain pedestrian reacquisition [3]. Accelerated by the exponential growth of CNNs [4–8], revolutionary breakthroughs have emerged in human pose estimation. For example, Newell’s stacked hourglass network (SHN) [4] utilizes staged feature extraction, feature fusion, and intermediate supervision techniques for human pose estimation. However, accurately localizing key points remains a significant challenge in situations where limbs overlap each other, or the human body is occluded by obstacles.

**Fig 1:**
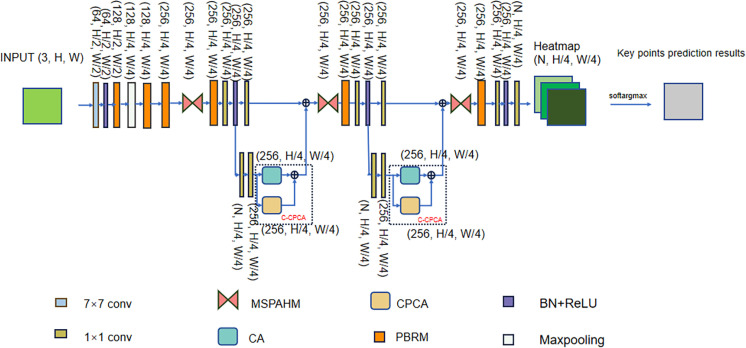
Overall model.

To address the aforementioned challenges, we draw inspiration from [9]. By introducing the attention module into the network, different regions of the human body’s key points can be represented with varying weights, leading to more discriminative representations. In the literature [10], channel prior convolutional attention (CPCA) was proposed as an attention mechanism. We integrate CPCA with Coordinate Attention (CA) [11] to form a new attention mechanism called coordinate – channel prior convolutional attention (C-CPCA), enabling the network to protect keypoints’ position information while enhancing the model’s ability to focus on key point areas. The C-CPCA is then incorporated into the network to guide subsequent hourglass modules in feature extraction, thereby improving pose estimation accuracy, locating the key points more accurately, and minimizing confusion in the complex scenarios. Furthermore, inspired by [12], we adopt its proposed module to enhance the hourglass module. Specifically, the multi-scale spatial pyramid attention (MSPA) module is used to replace the standard convolution in the hourglass module. This substitution allows the model to capture multi-scale spatial features with finer granularity and build extended dependencies across the channel. By stacking multiple multi-scale spatial pyramid attention hourglass modules (MSPAHMs), the model is better equipped to capture the structural dependencies between limb joints, thereby improving its ability to reason under scenarios where limbs are occluded or overlapped. In addition, the PinSK Bottleneck Residual Module (PBRM), which integrates Pinwheel-shaped Convolution (PConv) [13] and Selective Kernel Convolution (SKConv) [14], effectively improves multi-scale feature extraction by expanding the receptive field and dynamically adjusting the size of the convolution kernel, which enhances the network’s adaptability to complex poses and targets at different scales, thus significantly improving the accuracy of pose estimation. Based on these three advanced components, we propose a novel PoseNet++. PoseNet++ is illustrated in Fig 1. We verified the human pose estimation performance of PoseNet++ on four public datasets (MPII [15], LSP [16], COCO2017 [17], CrowdPose [18]). The results verify our network performance. Overall, the contributions of our research can be briefly summarized as the following four aspects:

1. We propose a novel multi-scale spatial pyramid attention hourglass module (MSPAHM) that enhances the model’s ability to capture multi-scale spatial features and extended dependencies across channels. This enables fine-grained local feature capture and global feature extraction with varying receptive fields, while also obtaining global dependencies between joints through channel interactions. Stacking MSPAHMs allows the model to better capture structural dependencies between limb joints, improving performance in occluded or overlapping scenarios.
2. We explore a C-CPCA mechanism by integrating CA and CPCA. The introduction of C-CPCA can enable the model to preserve keypoints’ location information and focus on keypoints’ regions, locating the keypoints more accurately and minimizing confusion between keypoints of the target person and people or sundries in the scenarios.
3. We propose a novel PinSK Bottleneck Residual Module (PBRM) that optimizes the receptive field and kernel selection, enhancing the network’s ability to extract features across multiple scales and complex poses, thereby improving pose estimation accuracy.
4. Compared with other mainstream human pose estimation models in recent years, PoseNet++ achieves the state-of-the-art performance on the MPII, LSP, COCO and CrowdPose datasets. At the same time, the model complexity of PoseNet++ is much lower than that of methods with similar accuracy.

## 2. Related work

### 2.1. Human pose estimation

HPE, regarded as an important topic within computer vision, aims to identify the key points on the human body within images or video frames. It has found broad usage in domains like augmented reality and action recognition. Despite significant progress, HPE remains a challenging problem, especially in cases of occlusion, complex scenarios, or diverse human poses. Multiple methods have been invented over the years, including early handcrafted models and modern deep learning approaches.

Traditional approaches, such as image structural modeling [16,19–21], depict the spatial relationships between limbs using tree-structured graphical models, which incorporate kinematic constraints to link the body parts. These approaches are based on handcrafted features, with examples like histograms of oriented gradients (HOG) [22]. However, their reliance on manually generated features for predicting keypoints’ locations makes them susceptible to challenges such as occlusion.

Benefiting from the swift advancements in deep learning techniques [23], increasingly efficient HPE approaches have also been introduced. Utilizing a convolutional neural network, the method proposed in [6] directly maps each keypoint of the human body to corresponding 2D pixel coordinates through regression-based prediction. Employing a convolutional neural network, the methods proposed in [4,7,24–26] produce probabilistic heatmaps through which human keypoints’ spatial positions are predicted. Among these, SHN [4] is particularly notable.

Building on the hourglass framework, various improvements have been proposed. SPCNet [27] incorporates Dilated Hourglass Modules (DHM) to enlarge the receptive field and Selective Information Modules (SIM) for better feature fusion in an 8-stack architecture. In [28], an affect module and residual attention mechanism are introduced to enhance resolution and focus on key regions, respectively, within a 2-stack network. In [29], the model proposed in that paper incorporates a range of lightweight strategies to achieve fast pose estimation while minimizing both computational demands and storage requirements. To optimize the encoder’s feature extraction process in each hourglass network, a shuffle-gated block is applied, effectively reducing parameter size and computation. In this paper, an hourglass network with a stacking number of 3 is chosen as the basic network.

### 2.2. Attention mechanism

HPE, which focuses on estimating the positions of key human body landmarks from visual data, is a core topic of computer vision. Despite the significant advancements brought by deep learning, accurately estimating poses in cases of occlusions, background clutter, and varying appearances continues to pose substantial challenges. In recent years, attention mechanisms have shown great promise in helping models selectively focus on critical spatial or semantic information, thus improving performance in various vision tasks. This motivates their integration into HPE frameworks to enhance feature representation and keypoint localization.

Attention mechanisms are designed to help models prioritize task-relevant regions or features. Common attention types include spatial attention, which emphasizes informative spatial regions, and channel attention, which highlights useful feature channels. While attention mechanisms have seen extensive application in domains like image recognition, their use in HPE is still relatively limited.

Chang et al. [30] introduced a model that incorporates HPE results as motion features and applied a channel-based attention mechanism to enhance spatial detail extraction. However, this approach exhibits limited effectiveness due to its insufficient modeling of inter-channel dependencies. Therefore, we enhance PoseNet++ by jointly incorporating spatial and channel attention modules, which helps the model extract critical features more effectively and boost the precision of keypoint localization.

## 3. Methodology

### 3.1. Overview of the PoseNet++

This study presents PoseNet++, an enhanced framework derived from SHN. To overcome SHN’s difficulty in capturing the structural relationship between limb joints and its poor performance under occlusion and complex poses, we introduce a lightweight hourglass module, MSPAHM. To mitigate the issue of human joints being easily confused with the targets in the complex scenarios, C-CPCA is proposed. To address the limitations of fixed receptive fields—which struggle to adapt to varying joint scales and misalign with target spatial distributions—we introduce a lightweight residual module, PBRM. Fig 1 illustrates the overall architecture. The three numbers in parentheses next to each module in the figure represent the dimensions (i.e., number of channels, height, and width) of the feature map output from that module. For example, (256, H/4, W/4) means that the feature map has 256 channels, the height is H/4, and the width is W/4. The dimensions of the input feature map are (3, H, W), where 3 represents the number of channels, and H and W represent the height and width, respectively. The input feature map is sequentially processed by the following modules:

1. Initial feature extraction: 7 × 7 convolutional layer, the output feature map size is (64, H/2, W/2); Batch Normalization and ReLU activation function processing, the output feature map size remains unchanged at (64, H/2, W/2); PBRM module, the output feature map PBRM module, the output feature map size is (128, H/2, W/2); Max Pooling, the output feature map size is (128, H/4, W/4); again through the PBRM module, the output feature map size is (128, H/4, W/4); another PBRM module, the output feature map size is (256, H/4, W/4); MSPAHM module which outputs feature maps of size (256, H/4, W/4).
2. Feature enhancement and branching processing: PBRM module, the output feature map size is (256, H/4, W/4); 1 × 1 convolutional layer, the output feature map size is (256, H/4, W/4); batch normalization and ReLU activation function processing, the output feature map size remains unchanged at (256, H/4, W/4). Subsequently, the feature map is divided into two parts: the first part passes through a 1 × 1 convolutional layer with an output feature map size of (256, H/4, W/4); the second part passes through an intermediate supervisory process, which consists of: a 1 × 1 convolutional layer to generate the heatmap, with a heatmap size of (N, H/4, W/4), where N denotes the number of keypoints; a 1 × 1 convolutional layer with an output feature map size of (256, H/4, W/4); C-CPCA module with an output feature map size of (256, H/4, W/4). The above two parts of the feature map are summed up by elements to get the fused feature map.
3. Further feature optimization and branching processing: MSPAHM module, the output feature map size is (256, H/4, W/4); PBRM module, the output feature map size is (256, H/4, W/4); 1 × 1 convolutional layer, the output feature map size is (256, H/4, W/4); batch normalization and ReLU activation function processing, the output feature map size remains unchanged at (256, H/4, W/4). Once again, the feature map is divided into two parts: the first part passes through a 1 × 1 convolutional layer, and the output feature map size is (256, H/4, W/4); the second part passes through an intermediate supervisory process, which consists of: a 1 × 1 convolutional layer to generate the heatmap, with the heatmap size of (N, H/4, W/4); a 1 × 1 convolutional layer, with the output feature map size of (256, H/4, W/4); a C-CPCA module which outputs feature maps of size (256, H/4, W/4). The above two parts of the feature map are summed up by elements to get the further optimized feature map.
4. Final feature processing and keypoint generation: MSPAHM module, the output feature map size is (256, H/4, W/4); PBRM module, the output feature map size is (256, H/4, W/4); 1 × 1 convolutional layer, the output feature map size is (256, H/4, W/4); batch normalization and ReLU activation function processing, the output feature map size remains unchanged at (256, H/4, W/4). Finally, the final heatmap is generated by 1 × 1 convolutional layer with heatmap size of (N, H/4, W/4) and the Softargmax function is applied to extract the key point coordinates.

### 3.2. Design scheme of the MSPAHM

#### 3.2.1. Revisit the original hourglass module.

For precise human pose estimation, it is essential that the network efficiently captures both local and global contextual information. Complex poses, occlusions, and varying image scales make human pose estimation tasks very difficult. To solve these problems, our approach employs SHN. This design allows the network to integrate features across different resolutions and continuously improve the accuracy of predictions.

**Fig 2:**
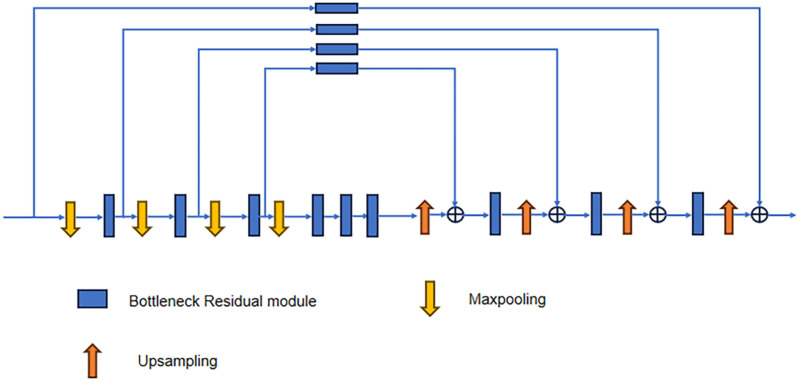
Structure of the original hourglass module.

**Fig 3:**
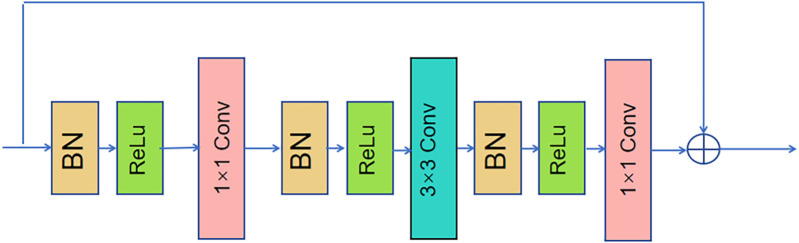
Structure of bottleneck residual module in the original hourglass module.

SHN is composed of multiple hourglass modules connected in series. The hourglass module’s structure is shown in Fig 2. Each hourglass module includes Maxpool layers, upsampling layers, and bottleneck residual modules. To maintain spatial information, the module extracts feature points at four different scales: the original size, half the original size, a quarter of the original size, and an eighth of the original size. After each pooling operation, the resolution of the feature map is reduced, leading to decreased computational complexity, while the bottleneck residual module helps in extracting image features. The feature map is then upsampled using nearest-neighbor interpolation to regain higher resolution. This process captures multi-scale and contextual information, preserving details from all layers while ensuring the image size remains unchanged. Consequently, the network retains the original image size without alteration. It can be clearly seen from Fig 2 that it employs the bottleneck residual module as the foundational element. The dual-path architecture of the bottleneck residual module is shown in Fig 3.

#### 3.2.2. Revisit the multi-scale spatial pyramid attention (MSPA).

**Fig 4:**
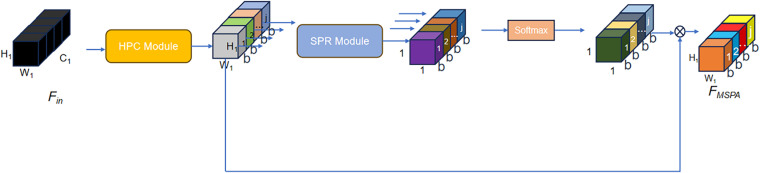
MSPA module.

In human pose estimation tasks, precise modeling of spatial features and semantic relationships across different body parts is crucial for accurate keypoint localization. However, conventional attention mechanisms often struggle to effectively capture multi-scale spatial dependencies, structural information, and long-range channel relationships within complex visual scenes. To overcome these challenges, we incorporate a lightweight and efficient module—MSPA [12]—that facilitates the feature representation of convolutional neural networks by incorporating fine-grained spatial details, hierarchical structural cues, and long-range channel dependencies. This module is particularly beneficial in human pose estimation, where both global context and local detail play complementary roles in refining predictions.It consists of three components: hierarchical phantom convolution (HPC), spatial pyramid recalibration (SPR), and Softmax. Its structure is shown in Fig 4. The following is the introduction to the internal process of MSPA.

The MSPA architecture processes input feature maps $F_{in}\in R^{C_{1}\times H_{1}\times W_{1}}$ , through sequential transformations. Here, $C_{1}$ $H_{1}$, and $W_{1}$ denote channel count, height, and width, respectively. The HPC module first divides $F_{in}$ channel-wise into j subsets $F_{a}\in R^{b\times H_{1}\times W_{1}}$, where channel dimensions satisfy $C_{1}=j\times b$. The feature subset $F_{a}$ is processed by hierarchical residual convolution, and the process can be expressed as the following mathematical formula:


$$F_{a}^{\prime }=\{\begin{array}{c} {}*20c\phi_{i}(F_{a}),a=1\\ \phi_{i}(F_{a}+F_{a-1}^{\prime }),a>1 \end{array}$$ (1)

where $\phi_{i}(\cdot )$ is the hierarchical residual convolution processing mentioned above, and + is element-by-element addition. $F_{a-1}^{\prime }$ is spliced into the output $F_{HPC}$, which can be expressed mathematically as:


$$F_{HPC}=Con\left(F_{1}^{\prime },F_{2}^{\prime },\ldots ,F_{j}^{\prime }\right)$$ (2)

where Con(·) is the splicing operation mentioned above.

Secondly, entering the SPR module, the feature map $F_{a}\in R^{b\times H_{1}\times W_{1}}$ undergoes global pooling and local average pooling operations to obtain all and local context features, respectively, and then these features are fused through an upsampling operation and in a weighted summation manner. Subsequently, the output is adjusted to a one-dimensional vector $G_{a}\in R^{4b\times 1\times 1}$.This process can be expressed mathematically as follows:


$$G_{a}=RS\left(a⊗UP\left(PL(F_{a}^{\prime },1)\right)+b⊗UP\left(PL(F_{a}^{\prime },2)\right)\right)$$ (3)

where RS(·) is an operation that adjusts the output into a one-dimensional vector. a and bare two learnable weight parameters. PL(·) is a pooling operation. ⊗ is element-wise multiplication. UP(·) is the upsampling operation. Pointwise convolution operation and sigmoid function are used to generate at weights $X_{a}$. This process can be expressed mathematically as follows:


$$X_{a}=\sigma (PCon\nu_{1}\left(ReLU\left(PCon\nu_{2}(G_{a})\right)\right)$$ (4)

where σ(·) is the sigmoid function. PConv1(·) and PConv2(·) are two point-wise convolutions. Functioning as the activation mechanism, the Rectified Linear Unit (ReLU(·)) introduces nonlinear transformations, while the channel-wise attention weights $X_{a}$ undergo probabilistic normalization via the Softmax operator, and this step can be expressed mathematically as follows:


$$\mathrm{\backslash [}Q_{a}=Softmax(X_{a})=\frac{\exp(X_{a})}{\sum_{v=1}^{j}\exp(X_{a})}\mathrm{\backslash ]}$$ (5)

Then, ${𝒬}_{a}$ is multiplied by $F_{a}^{\prime }$ respectively to obtain refined features ${\tilde{F}}_{a}$. This step can be expressed mathematically as follows:


$${\tilde{F}}_{a}=Q_{a}⊗F_{a}^{\prime }$$ (6)

Concatenate all ${\tilde{F}}_{a}$ to get $F_{MSPA}$. This step can be expressed mathematically as follows:


$$F_{MSPA}=Con\left({\tilde{F}}_{1},{\tilde{F}}_{2},...,{\tilde{F}}_{j}\right)$$ (7)

#### 3.2.3. Propose the multi-scale spatial pyramid attention hourglass module (MSPAHM).

**Fig 5:**
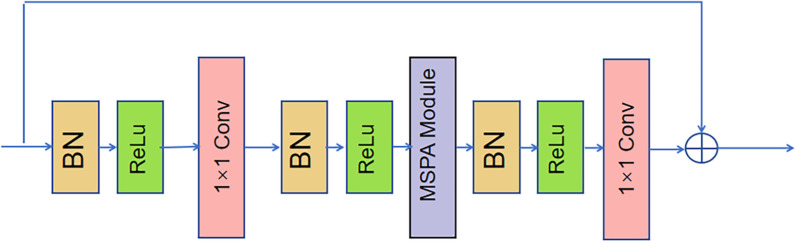
The enhanced bottleneck residual module in the MSPAHM.

Recent studies have shown that incorporating attention mechanisms into Convolutional Neural Networks (CNNs) can significantly enhance network performance. Notably, mechanisms such as Squeeze-and-Excitation Networks (SENet) [31], Convolutional Block Attention Module (CBAM) [32] and Coordinate Attention (CA) [11] have demonstrated substantial improvements. Meanwhile, the structure of the SHN contains an excessive number of bottleneck residual modules, resulting in a large network and increased redundancy in data processing. As illustrated in Fig 3, each bottleneck residual module requires one 3 × 3 standard convolution and two 1 × 1 convolutions. Given that the input images for the human pose estimation task are high-resolution, the standard convolution introduces a significant number of parameters and computations, which decreases the model’s training efficiency. Additionally, standard convolution lacks cross-channel information exchange, but cross-channel information exchange is especially important for HPE. Therefore, this paper employs a lightweight and efficient MSPA module to substitute the 3 × 3 convolution in the bottleneck structure of the hourglass module, as shown in Fig 5.

The input feature map is first processed by BN+RELU and then enters into the first 1 × 1 convolution process. The number of channels is reduced to half of its original value. After another BN+RELU processing, the feature map enters into MSPA module processing to capture multi-scale spatial features and extended dependencies across channels. The number of channels remains unchanged. After the final BN+RELU processing, the feature map enters into the 1 × 1 convolution. The number of channels becomes the same as that of the original input feature map. The feature map outputted by the 1 × 1 convolution is added element-wise to the original input feature map to get the final output feature map. Compared with the direct use of the 3 × 3 standard convolution, MSPAHM enhances the model’s ability to capture multi-scale spatial features with finer granularity and build extended dependencies across channels, thereby capturing fine-grained local features (such as small joints) and global features (such as the overall limb structure) with different receptive fields and obtaining global dependencies between different joints by interacting along the channel axis of the feature tensor. By stacking multiple MSPAHMs, the model is better equipped to capture the structural dependencies between limb joints, thereby improving its ability to reason under scenarios where limbs are occluded or overlapped.

### 3.3. Design scheme of the C-CPCA module

#### 3.3.1. Revisit the coordinate attention (CA).

**Fig 6:**
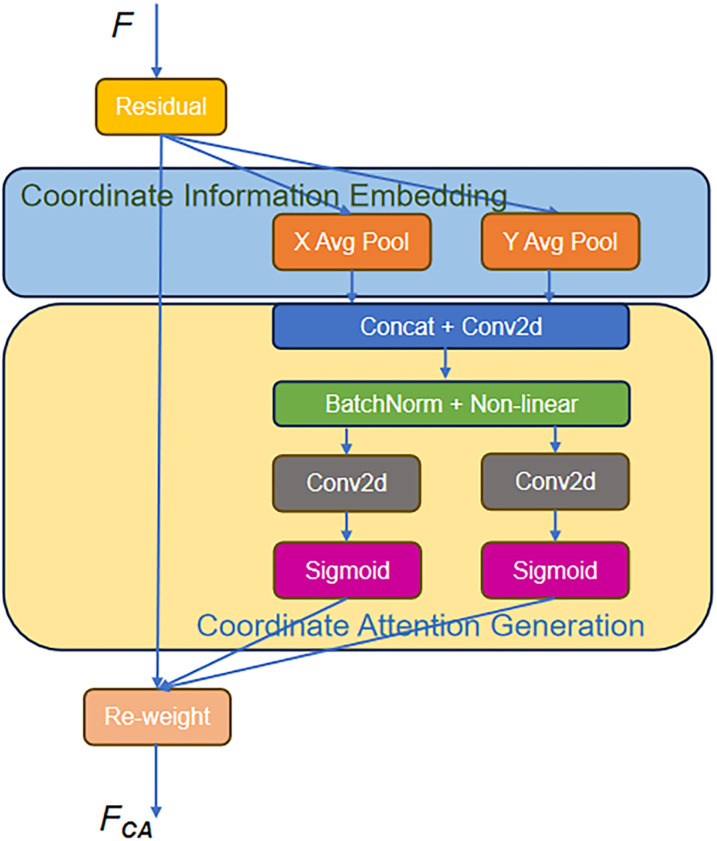
Coordinate attention.

To improve the accuracy of HPE, especially under occlusions and in complex multi-person scenarios, this paper incorporates CA [11] into SHN. Traditional attention modules (e.g., SE, CBAM) either focus only on inter-channel relationships or compress spatial information. In contrast to these modules, CA introduces a lightweight and efficient way to encode channel dependencies and positional information by decomposing global pooling into two one-dimensional operations along the horizontal and vertical directions. CA proves highly effective in HPE because of its ability to capture long-range contextual information while retaining precise spatial locations. Fig 6 illustrates the architecture of CA, followed by an explanation of how the feature map is processed through the CA module.

The input feature map $F\in R^{C_{a}\times H_{a}\times W_{a}}$, where $W_{a}$, $H_{a}$ and $C_{a}$ represent the width, height, and number of channels, respectively, undergoes a two-step transformation. In the first step, the input feature map is encoded along the horizontal and vertical directions using global pooling. Specifically, for the c-th channel at height h, the encoded feature is given by:


$$\mathrm{\backslash [}z_{h}^{c}(h)=\frac{1}{W_{a}}\sum_{i=0}^{W_{a}-1}x^{c}(h,i)\mathrm{\backslash ]}$$ (8)

where $x^{c}(h,i)$ refers to the feature at position (h,i) in the c-th channel, and $W_{a}$ is the width of the tensor. Similarly, for the c-th channel at width w, the encoded feature is:


$$\mathrm{\backslash [}z_{w}^{c}(w)=\frac{1}{H_{a}}\sum_{j=0}^{H_{a}-1}x^{c}(\;j,w)\mathrm{\backslash ]}$$ (9)

These two features are then concatenated and passed through a 1 × 1 convolutional layer to capture the integrated spatial information, yielding an intermediate feature map $f\in R^{\frac{c_{a}}{r}\times (H_{a}+W_{a})}$, where r is a reduction ratio. The next step in the CA mechanism involves generating the coordinate attention maps. The intermediate feature map f is split into two parts: $f_{h}$ for the horizontal direction and $f_{w}$ for the vertical direction. Each of these parts is transformed via separate 1 × 1 convolutional layers, generating attention maps $g_{h}$ and $g_{w}$ respectively:


$$g_{h}=\sigma \left(F_{h}(f_{h})\right),\;g_{w}=\sigma \left(F_{w}(f_{w})\right)$$ (10)

where $F_{h}$ and $F_{w}$ are 1 × 1 convolutional transformations. The final output $F_{CA}$ is obtained by applying these attention maps to the original input feature map:


$$F_{CA}=y^{c}(i,j)=x^{c}(i,j)⊗g_{h}^{c}(i)⊗g_{w}^{c}(j)$$ (11)

#### 3.3.2. Revisit the channel prior convolutional attention (CPCA).

**Fig 7:**
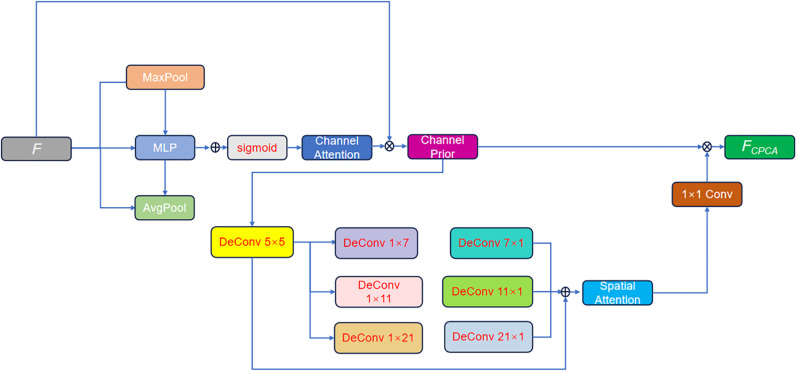
CPCA module.

HPE seeks to detect key joint positions within images or video frames. Nevertheless, traditional methods struggle to capture complex spatial relationships. As a result, they have difficulty distinguishing important features in complex scenarios. We incorporate CPCA [10] to respond to these challenges. CPCA fuses channel attention with spatial attention. Incorporating CPCA enables our model to dynamically adjust its focus on the most significant feature channels and spatial regions rich in information. This dynamic allocation of attention in both dimensions is crucial for improving the model’s capacity to capture fine-grained details in HPE. A schematic of its architecture is illustrated in Fig 7, and the processing flow of the feature map within CPCA is subsequently described.

The feature map $F\in R^{C_{a}\times H_{a}\times W_{a}}$ undergoes global average pooling (GAP) and global maximum pooling (GMP) to obtain two channel identifiers $F_{avg}$ and $F_{\max}$. This process can be expressed as:


$$F_{avg}=GAP(F),F_{max}=GMP(F)$$ (12)

$F_{avg}$ and $F_{\max}$ are input to a shared multi-layer perceptron (MLP), and the sigmoid activation function is used to calculate the channel attention map. This process can be expressed as:


$$M_{ch}=\sigma \left(MLP\left(F_{avg}\right)+MLP(F_{max})\right)$$ (13)

The channel attention $M_{sp}$ is then applied to the feature map F by element-by-element multiplication. This process can be expressed as:


$$F_{ch}=M_{ch}⊗F$$ (14)

where $F_{ch}$ is the feature map obtained by channel attention weighting. $F_{ch}$ is input to the spatial attention module for processing. Capture spatial information through a multi-scale depth convolution module, generate intermediate spatial feature representations, and then fuse multi-scale spatial features through summation. Then a 1 × 1 convolution operation is applied to the fused feature to further integrate inter-channel information and generate the final spatial attention weight map. This process can be summarily expressed as:


$$\mathrm{\backslash [}M_{sp}=\sigma \left(Conv{\mathrm{\backslash nolimits}}_{1\times 1}\left(\sum_{i=1}^{K}DwConv_{k_{i}}\left(F_{ch}\right)\right)\right)\mathrm{\backslash ]}$$ (15)

where $M_{sp}$ is the generated spatial attention map. Finally, the output feature map is obtained by multiplying $F_{ch}$ and $M_{sp}$ element by element: $Conv_{1\times 1}$ represents the 1 × 1 convolution of channel mixing. $DwConv_{k_{i}}$ is the i-th depth convolution with kernel ki×ki. Finally, the output feature map is obtained by multiplying $F_{ch}$ and $M_{sp}$ element by element:


$$F_{CPCA}=M_{sp}⊗F_{ch}$$ (16)

where $F_{CPCA}$ is the final output feature map.

#### 3.3.3. Propose the coordinate-channel prior convolutional attention (C-CPCA).

**Fig 8:**
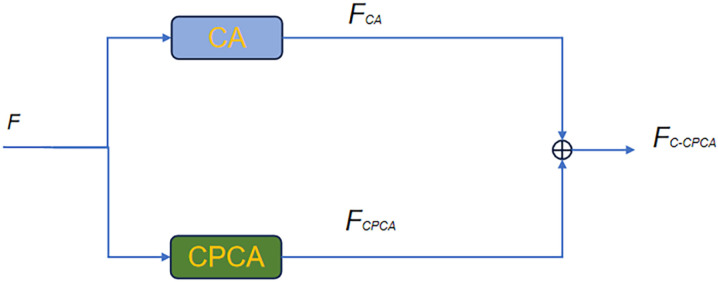
C-CPCA module.

In complex scenarios, such as scenes involving multiple people or sundries, the joints of the target person are easily confused with targets in the scenarios. C-CPCA integrates coordinate attention (CA) and channel prior convolutional attention (CPCA) to preserve keypoints’ location information and focus on keypoints’ regions, locating the keypoints more accurately and minimizing confusion between keypoints of the target person and people or sundries in the scenarios. The structure of C-CPCA is shown in Fig 8.

The input feature map $F\in R^{C_{a}\times H_{a}\times W_{a}}$ is input into the C-CPCA module, where it is processed separately by both Coordinate Attention (CA) and Channel Prior Convolutional Attention (CPCA) to obtain the refined features. $F_{CA}$ and $F_{CPCA}$, respectively. Finally, the output feature $F_{C-CPCA}$ is generated by performing element-wise addition of $F_{CA}$ and $F_{CPCA}$. The C-CPCA can be expressed mathematically as follows:


$$F_{C-CPCA}=F_{CA}+F_{CPCA}$$ (17)

The C-CPCA module is inserted into SHN at the position indicated by the dashed box in Fig 1. It is evident that the heat map generated by the preceding hourglass module is restored and processed within the C-CPCA module. The salient features in the feature maps are enhanced, leading to improved discrimination among the features. Consequently, it is anticipated that the desired performance in HPE can be achieved.

### 3.4. Design scheme of the PinSK bottleneck residual module

#### 3.4.1. Revisit the selective kernel convolution (SKConv).

HPE has emerged as a significant topic within computer vision. Traditional methods often struggle to cope with challenges such as target scale variation, occlusion and complex scenarios. SKConv [14] is incorporated to address these problems. SKConv improves HPE by incorporating adaptive receptive field (RF) adjustments. The incorporation of SKConv enables PoseNet++ to dynamically adjust its RF in response to the scale and complexity of the input features. Our model improves its capacity to localize human joints more precisely under different conditions. This selective kernel mechanism is particularly important for handling multi-scale and occluded human body regions. SKConv is a computationally efficient component that adds minimal overhead to the overall architecture. The processing flow of the feature map within SKConv is subsequently described.

**Fig 9:**
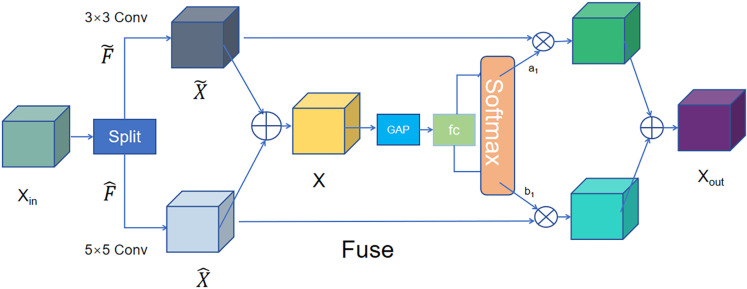
SKConv module.


As shown in Fig 9, there are three processes within SKConv: Split, Fuse, and Select. The split phase is performed by employing 3 × 3 and 5 × 5 multi-branch convolutional pairs $X_{in}\in R^{C_{2}\times H_{2}\times W_{2}}$, where $C_{2}$, $H_{2}$, and $W_{2}$ represent the number of channels, height, and width, respectively. The mathematical expression of this process is as follows:

$$\tilde{X}=\tilde{F}(X),\;\hat{X}=\hat{F}(X)$$ (18)

where $\tilde{X}$ and $\hat{X}\;$denote convolutional operations using different kernel sizes.

The Fuse operation combines the information from these multiple paths through element-wise summation, forming a fused feature map X. Its function expression is shown as follows:


$$X=\tilde{X}+\hat{X}$$ (19)

The channel-level statistic s is extracted by Global Average Pooling. Its function expression is shown as follows:


$$\mathrm{\backslash [}s=\frac{1}{H_{2}\times W_{2}}\sum_{i=1}^{H_{2}}\sum_{j=1}^{W_{2}}X(i,j)\mathrm{\backslash ]}$$ (20)

Further, a compact feature is created to enable the guidance for the precise and adaptive selections. This is achieved by a simple fully connected (fc) layer. Subsequently, the Select operation employs a softmax attention mechanism to assign different weights for selecting information from various kernel sizes, resulting in the final output $X_{out}$. Its function expression is shown as follows:


$$X_{out}=a_{1}\cdot \tilde{X}+b_{1}\cdot {\hat{X}}_{c}$$ (21)

where the weights $a_{1}$ and $b_{1}$ are dynamically generated based on the input information, guiding the flow of information from different convolutional kernels.

#### 3.4.2. Revisit the pinwheel-shaped convolution (PConv).

**Fig 10:**
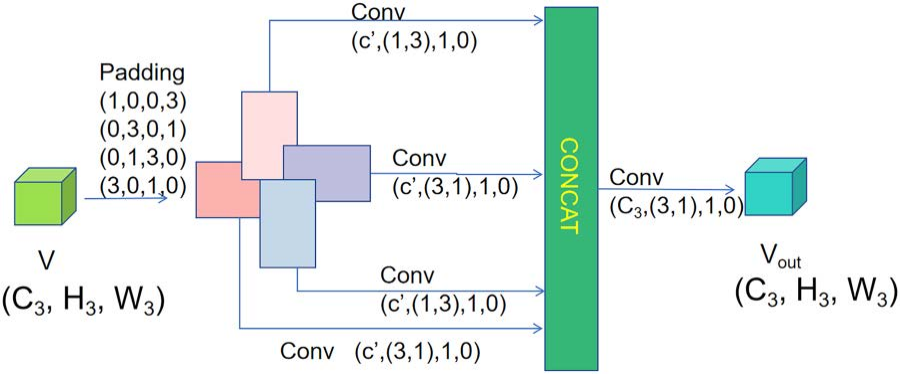
PConv module.

HPE represents a fundamental problem in computer vision, with the objective of precisely determining the positions of human keypoints. However, existing convolutional neural network (CNN) methods often face challenges in extracting underlying features and expanding the RF when handling complex human poses. To address these issues, we incorporate Pinwheel-shaped Convolution (PConv) [13]. PConv significantly expands the RF of PoseNet++ and improves the underlying feature extraction capability through asymmetric padding and multi-directional convolution kernels. In addition, PConv is a lightweight module that maintains model efficiency without introducing substantial computational burden. The structure of PConv is illustrated in Fig 10. The following is the introduction to the internal process of PConv.

The input feature map $V\in R^{C_{3}\times H_{3}\times W_{3}}$ is subjected to four sets of convolution operations in different directions, and each set of outputs is processed by Batch Normalization (BN) and SILU activation function, where $W_{3}$, $H_{3}$, and $C_{3}$ represent the width, height, and number of channels, respectively. Its function expression is shown as follows:


$$\begin{array}{c} {}*20cV_{1}=SiLU\left(BN\left(V_{P(1,0,0,3)}⊗Q_{1}^{\left(1,3,c^{\prime }\right)}\right)\right),\\ V_{2}=SiLU\left(BN\left(V_{P(0,3,0,1)}⊗Q_{2}^{\left(3,1,c^{\prime }\right)}\right)\right),\\ V_{3}=SiLU\left(BN\left(V_{P(0,1,3,0)}⊗Q_{3}^{\left(1,3,c^{\prime }\right)}\right)\right),\\ V_{4}=SiLU\left(BN\left(V_{P(3,0,1,0)}⊗Q_{4}^{\left(3,1,c^{\prime }\right)}\right)\right). \end{array}$$ (22)

where BN(·) represents the batch normalization operation. SiLU(·) represents the sigmoid-weight linear unit activation function. $V_{P(a,b,c,d)}$ represents the tensor after the input feature diagram is filled with asymmetric filling and (a, b, c, d) denotes the number of filled pixels in the left, right, top, and bottom directions, respectively. $Q_{i}^{\left(k_{h},k_{w},c^{\prime }\right)}\;$represents the weight matrix of the convolution kernel of group i. $k_{h}\;\times \;k_{w}$ represents the kernel size. $c^{\prime }$represents the number of output channels. Then, the four sets of feature maps are spliced along the channel dimensions and further fused through 2 × 2 convolution. Its function expression is shown as follows:


$$V_{out}^{\left(H_{3},W_{3},C_{3}\right)}=SiLU\left(BN\left(Con(V_{1},\ldots ,V_{4})⊗U^{\left(2,2,C_{3}\right)}\right)\right)$$ (23)

where $U^{\left(2,2,C_{3}\right)}$ represents the fusion convolutional kernel with size 2 × 2 and number of output channels $C_{3}$.

#### 3.4.3. Propose the PinSK bottleneck residual module (PBRM).

**Fig 11:**
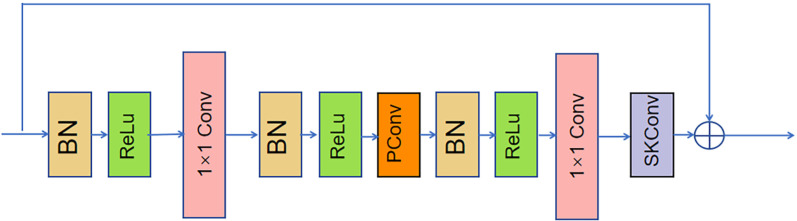
PBRM module. After the input feature map is processed by BN+RELU, it enters the first 1 × 1 convolution. The number of channels becomes half of the original. After another BN + ReLU processing, the feature map enters PConv to expand the receptive fields to better match the hierarchical arrangement of human skeletal features. After the BN+RELU processing, the second 1 × 1 convolution process is performed. The number of feature channels becomes the same as the number of the original input channels. Then the feature map enters SKConv for processing to make dynamic kernel hanging according to the input characteristics to optimize multi-scale feature processing, and the number of feature map channels remains unchanged. Finally, the feature map outputted by SKConv is added element-wise to the input feature map to get the output feature map. By introducing PBRM, which addresses the limitations of RF and scale adaptation, the model achieves more accurate estimation of complex human poses.

SHN has demonstrated promising performance in human pose estimation; however, it still faces several limitations, particularly regarding the receptive field size and its capacity to handle multi-scale features effectively. The bottleneck residual blocks in the baseline architecture utilize standard 3 × 3 convolutions, which efficiently capture local features but face limitations in representing large-scale, intricate human pose variations and fine-grained joint motions. A key constraint arises from the fixed kernel dimensions, restricting adaptability to diverse anatomical configurations. To address these issues, we improve the remaining bottleneck residual modules in the network except for the hourglass module. Specifically, we replaced 3 × 3 convolution with PConv and added SKConv after the second 1 × 1 convolution. We named the novel bottleneck residual module PinSK Bottleneck Residual Module (PBRM) and its structure is shown in Fig 11.

### 3.5. Ethics statement

Ethical approval and patient consent for the study were obtained for the MPII [15], LSP [16], COCO2017 [17], and CrowdPose [18] datasets. No human participants were recruited specifically for this study. We didn’t report a retrospective study of medical records or archived samples. All datasets utilized (MPII, LSP, COCO2017, and CrowdPose) are publicly available and have been extensively adopted in previous researches [4,27–29] related to human pose estimation.

## 4. Results

### 4.1. Datasets

This paper evaluates PoseNet++ in four commonly used human pose data sets (MPII [15], LSP [16], COCO2017 [17] and CrowdPose [18]). The MPII dataset contains about 25,000 images, including 40,000 samples containing 16 key points. 28,000 samples are divided into the training set, and 11,000 samples are divided into the test set. According to the method of [4] literature, 3,000 samples are allocated as a verification set for hyperparameter adjustment. Notably, MPII is primarily composed of daily activity scenes featuring upright and frontal poses, which may introduce a pose distribution bias, potentially limiting the generalization to more diverse or complex poses in real-world settings.

This paper collectively refers to the LSP original data set and the extended data set as the LSP data set, which contains 11,000 samples divided into the training set and 1,000 samples divided into the test set. It mainly includes sports-related images with exaggerated and highly articulated body configurations. While LSP helps models learn flexible pose structures, it lacks the diversity of clothing and backgrounds, which may affect performance in everyday or multi-person scenarios.

The COCO2017 dataset contains more than 200,000 images, annotated with 250,000 samples containing 17 joint nodes, 57,000 images are divided into the training set, 5,000 images are divided into the verification set, and 20,000 are divided into the test set. Despite its scale and variety, COCO has relatively fewer extreme or rare poses compared to datasets like LSP, and some images include crowd occlusions and truncated people that pose additional challenges.

The CrowdPose dataset contains 20,000 images, annotated with 80,000 samples containing 14 nodes, of which 10,000 images are divided into the training set, 2,000 images are divided into the verification set, and 8,000 are divided into the test set. This dataset introduces more complex pose estimation challenges arising from individual overlaps and visual obstructions, offering a complementary benchmark for evaluating robustness in crowded environments.

In summary, although these datasets together provide a comprehensive benchmark, they each exhibit specific biases—pose-type bias in MPII, activity bias in LSP, instance density bias in COCO, and occlusion bias in CrowdPose. These limitations can influence the generalization ability of models trained on them. To mitigate these effects, future work could incorporate synthetic data augmentation, domain adaptation techniques, or leverage more balanced datasets that capture broader real-world diversity.

### 4.2. Evaluation metrics

On MPII and LSP, this paper uses “Percentage of Correct Keypoints” (PCK) as the core evaluation index [15]. PCK calculates whether the spatial proximity between the detected joint and the marked truth value satisfies the ratio of preset thresholds, and is used to quantify the registration accuracy. For the MPII dataset, a normalized variant of PCK is used in this paper, that is, a part of the head size is used as a normalization factor, denoted as PCKh. In contrast, the evaluation of the LSP dataset follows the original PCK formula specified in the literature [33,34] to ensure consistency with previous studies. On the COCO2017 and CrowdPose datasets, this paper uses Average Precision (AP) and Average Recall (AR) calculated based on object keypoint similarity (OKS) as evaluation indicators. These indicators can fully reflect the model’s performance in multi-scale and multi-pose scenarios.

### 4.3. Implementation details and training settings

The PyTorch framework was utilized to implement the network architecture. The software platform utilized was Python 3.8. Training was conducted on a single NVIDIA GeForce RTX 3090 GPU, using a batch size set to 16. We trained our model using the Adam optimizer. The optimizer was configured with an initial learning rate of 5 × 10 ⁻ ⁴. To facilitate stable convergence, we applied a two-stage learning rate schedule. First, a linear warm-up was applied over the initial 500 iterations, during which the learning rate increased from 0.001 × the initial value (i.e., from 5 × 10 ⁻ ⁷ to 5 × 10 ⁻ ⁴). After the warm-up, a multi-step decay schedule was used: the learning rate was reduced by a factor of 0.1 at epoch 170 and again at epoch 200. During the training process, the widely utilized data augmentation techniques referenced in prior work [35] were adopted to reduce the risk of overfitting, including the following: (1) random scaling with factors ranging from 0.5 to 1.5; (2) random rotations between −80° and 80°; and (3) random horizontal flips applied with a 50% probability. Each image was rotated and scaled once during training, with specific parameters randomly sampled from the ranges mentioned above. Regarding the data preprocessing stage, each detected human bounding box was adjusted to maintain a constant 4:3 width-to-height ratio. The bounding boxes were utilized to crop the images accordingly. The image size of COCO2017 and CrowdPose datasets was set to 256 × 192 or 384 × 288, and the image size of MPII and LSP datasets was set to 256 × 256. We utilized the mean square error (MSE) as the loss function.

### 4.4. Ablative analysis

We conducted a systematic evaluation of the MSPAHM, C-CPCA Attention Mechanism, and PBRM by progressively integrating these components into the baseline architecture. Performance gains from integrating these enhancements are detailed in Table 1. A clear representation of the change in performance before and after module removal can be presented in Table 2. According to the first row of Table 2, when using the original hourglass module containing the standard bottleneck residual module, the number of model parameters (#Params) increased from 9.4M to 18.9M, the Giga Floating-Point Operations (GFLOPs) increased from 4.6 to 9.1, and the PCKh score decreased from 91.1 to 89.8. This result confirmed that the use of MSPAHM can effectively reduce model complexity. This result also demonstrated that MSPAHM enhances the model’s ability to capture multi-scale spatial features and construct extended dependencies across channels, thus enabling the capture of local features and global features at a finer granularity, and the acquisition of global dependencies across joints through the channel-axis interaction of the feature tensor. Therefore, with the help of MSPAHM, the accuracy of HPE becomes higher. The second row of Table 2 shows that, when C-CPCA was removed, #Params dropped by only 1.0M, GFLOPs decreased by only 0.7, but the PCKh score decreased by 1.7. This change proved that C-CPCA significantly optimizes the model’s localization accuracy, multi-scale perception and dynamic adaptation ability without significantly increasing the model complexity. The third record in Table 2 reveals that incorporating the traditional bottleneck residual module into the network raised #Params from 9.4M to 15.2M and increased GFLOPs from 4.6 to 6.0, accompanied by a reduction in the PCKh score from 91.1 to 90.7. This change confirmed the significant reduction in model complexity due to the removal of the 3 × 3 convolution in the residual module in favor of two lightweight convolutions (PConv and SKConv). This change also confirmed that the PBRM addresses the issues of constrained receptive fields and scale adaptation, allowing the model to more accurately estimate a wide range of complex human poses.

**Table 1:** Ablation experiment results (PCKh@0.5).

Method	MSPAHM	C-CPCA	PBRM	#Params(M)	GFLOPs	Mean
I	×	×	×	23.7	9.8	88.2
II	√	×	×	14.2	5.3	88.7
III	×	√	×	24.7	10.5	88.9
IV	×	×	√	17.9	8.4	89.0
V	√	×	√	8.4	3.9	89.4
VI	×	√	√	18.9	9.1	89.8
VII	√	√	×	15.2	6.0	90.7
VIII	√	√	√	9.4	4.6	**91.1**

**Table 2:** Clear representation of the change in performance before and after module removal (PCKh@0.5).

Method	#Params(M)	GFLOPs	Mean
w/o MSPAHM	18.9	9.1	89.8
w/o C-CPCA	**8.4**	**3.9**	89.4
w/o PBRM	15.2	6.0	90.7
Ours(PoseNet++)	9.4	4.6	**91.1**

### 4.5. Quantitative result

#### 4.5.1. Results on the MPII dataset.

In this section, the performance of PoseNet++ is analyzed in comparison with other efficient human pose estimation networks in recent years on the MPII dataset. Table 3 demonstrates the PCKh scores of PoseNet++ with other methods on the MPII validation set and with a threshold of 0.5. As can be seen from the table, the PCKh score of PoseNet++ improves by 2.9 compared to the original network SHN. Compared to the most recent (2024) method, EdgePose, the PCKh score of PoseNet++ is higher by 0.7. Compared to the previous method proposed by Tang et al., which previously had the highest PCKh score, the PCKh score of PoseNet++ is 0.6 higher. The table also lists the performance of transformer-based approaches for MPII validation set, such as TokenPose and SimCC. PoseNet++ has a PCKh score that is 0.9 higher than TokenPose and 1.1 higher than SimCC. Meanwhile, PoseNet has the highest localization accuracy at all keypoints. Table 4 demonstrates the PCKh scores of PoseNet++ versus other methods on the MPII test set with a threshold of 0.5. As can be seen from the table, the PCKh score of PoseNet++ improves by 3.2 compared to the original network SHN. Compared to the latest (2024) method proposed by Liu et al., the PCKh score of PoseNet++ is higher by 0.4. Compared to HRNet-W32, which had the highest PCKh score before, the PCKh score of PoseNet++ is higher by 0.1. Meanwhile, PoseNet++ has the highest localization accuracy at all key points except the wrist.

**Table 3:** Comparative experiments on the MPII validation set (PCKh@0.5).

Method	Head	Shoulder	Elbow	Wrist	Hip	Knee	Ankle	Mean
SHN [4]	96.6	95.3	88.4	83.4	88.3	82.6	78.3	88.2
Yang et al. [33]	97.4	96.2	91.1	86.9	90.1	86.0	83.9	90.3
Tang et al. [34]	97.4	96.2	91.0	86.9	90.6	86.8	84.5	90.5
AL [36]	96.5	96.0	90.5	86.0	89.2	86.8	83.7	89.9
HRNet-W32 [37]	97.1	95.9	90.3	86.4	89.1	87.1	83.3	90.3
PGCN [38]	–	–	–	83.6	–	–	80.8	88.9
UDP-Pose [39]	97.4	96.0	91.0	86.5	89.1	86.6	83.3	90.4
TokenPose [40]	97.1	95.9	90.4	86.0	89.3	87.1	82.5	90.2
Zhou et al. [41]	97.3	96.0	91.1	86.8	89.3	87.1	83.3	90.6
Tian et al. [42]	97.1	95.9	90.4	85.1	89.1	85.8	81.5	89.8
SimCC [43]	97.2	96.0	90.4	85.6	89.5	85.8	81.8	90.0
EdgePose [44]	–	–	–	–	–	–	–	90.4
Ours(PoseNet++)	**97.8**	**96.8**	**91.9**	**87.9**	**90.7**	**87.7**	**84.6**	**91.1**

**Table 4:** Comparative experiments on the MPII test set (PCKh@0.5).

Method	Head	Shoulder	Elbow	Wrist	Hip	Knee	Ankle	Mean
SHN [4]	96.7	95.7	89.9	84.8	88.1	84.3	81.0	89.2
Yang et al. [33]	98.5	96.7	92.5	88.7	91.1	88.6	86.0	92.0
Tang et al. [34]	97.4	96.2	91.0	86.9	90.6	86.8	84.5	90.5
Luvizon et al. [45]	98.1	96.6	92.0	87.5	90.6	88.0	82.7	91.2
HRNet-W32 [37]	98.6	96.9	92.8	**89.0**	91.5	89.0	85.7	92.3
EMpose [46]	98.3	96.6	91.9	87.8	90.5	88.2	84.4	91.4
HR-ARNet [47]	98.3	96.7	92.4	88.5	90.4	88.3	84.4	91.6
GLCFBNet [48]	98.4	96.7	92.2	88.0	91.2	88.9	84.8	91.8
Zhou et al. [49]	97.4	95.3	89.6	84.9	89.1	85.6	82.4	89.6
Liu et al. [50]	98.4	96.7	92.8	88.6	91.1	89.3	84.1	92.0
Ours(PoseNet++)	**98.6**	**97.0**	**92.9**	88.6	**91.6**	**89.5**	**86.3**	**92.4**

#### 4.5.2. Results on the LSP dataset.

This section compares this paper’s approach with existing methods on the LSP dataset using the evaluation metrics used in previous studies [33,34]. To ensure consistency with the existing literature [33], this paper adds MPII training data to the extended LSP training examples and evaluates the performance under these unified experimental conditions. As shown in Table 5, PoseNet++ outperforms all previous methods with 95.5 in terms of PCK score with a threshold of 0.2. Compared to the most recent (2021) and highest PCK score proposed by Tian et al., the PCK score of PoseNet++ is higher by 0.4. In addition, PoseNet++ outperforms all previous methods in the localization of keypoints such as the head, shoulder, elbow, and knee, achieving state-of-the-art (SOTA) performance.

**Table 5:** Comparative experiments on the LSP test dataset (PCK@0.2).

Method	Head	Shoulder	Elbow	Wrist	Hip	Knee	Ankle	Mean
Belagiannis et al. [51]	95.2	89.0	91.5	77.0	93.7	87.0	82.8	85.2
Chu et al. [52]	98.1	93.7	89.3	86.9	93.4	94.0	92.6	92.5
Yang et al. [33]	98.3	94.5	92.2	88.9	94.4	95.0	93.7	93.9
Ning et al. [53]	98.1	94.4	91.8	89.3	94.7	95.0	93.5	93.9
Chou et al. [54]	98.2	94.9	92.2	89.5	94.2	95.0	94.1	94.0
Tang et al. [34]	98.3	95.9	93.5	90.7	95.0	96.6	**95.7**	95.1
Lu et al. [55]	97.6	94.2	89.0	83.8	**96.3**	94.1	90.8	92.2
Tian et al. [42]	97.8	95.5	94.4	**92.9**	94.7	95.6	94.3	95.1
Ours(PoseNet++)	**98.3**	**96.1**	**94.4**	91.4	95.3	**96.7**	95.6	**95.5**

#### 4.5.3. Results on the COCO2017 dataset.

In this section, PoseNet++ is compared with other recent years’ efficient human pose estimation networks on the COCO2017 dataset for comparative analysis. Table 6 demonstrates the performance of PoseNet++ with other methods on the COCO val2017 set. At an input resolution of 256 × 192, PoseNet++ has a 75.4 AP and an 80.8 AR. Compared to the most recent (2024) method proposed by Liu et al., the AP of PoseNet++ is higher by 0.3, while the AR is higher by 0.6. Compared to GLCFBNet, which had the highest AP before, the AP of PoseNet++ is higher by 0.1, while AR is higher by 0.3. The table also lists the performance of transformer-based approaches in the COCO val2017 set, such as Tokenpose and SimCC. Compared to TokenPose, the AP of PoseNet++ is higher by 0.7, while AR is higher by 0.8. Compared to SimCC, the AP of PoseNet++ is higher by 1.8, while AR is higher by 1.9. At an input resolution of 384 × 288, PoseNet++ has a 76.5 AP and an 81.6 AR. Compared to the latest (2024) method proposed by Liu et al. PoseNet++ has 0.1 higher AP and, at the same time, 0.1 higher AR. Table 7 demonstrates how PoseNet++ compares to other methods in the performance on the COCO test-dev2017 set. At an input resolution of 256 × 192, PoseNet++ has a 74.7 AP and a 79.9 AR. Compared to the most recent (2024) method proposed by Liu et al. the AP of PoseNet++ is higher by 0.5, while the AR is higher by 0.6. Compared to GLCFBNet, which had the highest AP before, the AP of PoseNet++ is higher by 0.1, while AR is 0.1 higher. Compared to TokenPose, which is the transformer-based approach, the AP of PoseNet++ is higher by 0.7, while AR is higher by 0.8. At an input resolution of 384 × 288, PoseNet++ has a 75.5 AP and an 80.6 AR. Compared to the most recent (2024) and highest AP method proposed by Liu et al. PoseNet++ has a higher AP by 0.1 and a higher AR by 0.3.

**Table 6:** Comparisons on the COCO val2017 set.

Method	Backbone	Input Size	AP(%)	AR(%)
SHN [4]	SHN	256 × 192	66.9	–
HRNet [37]	HRNet-W32	256 × 192	74.4	79.8
OASNet [56]	HRNet-W32	256 × 192	75.0	80.4
HR-ARNet [47]	HRNet-W32	256 × 192	74.9	80.3
EMpose [46]	HRNet-W32	256 × 192	75.0	80.2
TokenPose [40]	TokenPose-B	256 × 192	74.7	80.0
SimCC [43]	TokenPose-S	256 × 192	73.6	78.9
FEM&MSFF [57]	ResNet-101	256 × 192	72.5	–
MSPENet [58]	ResNet-50	256 × 192	72.7	78.3
HPnet [59]	ResNet-152	256 × 192	73.7	–
DLIFP [60]	HRNet-W32	256 × 192	75.0	–
GLCFBNet [48]	SHN	256 × 192	75.3	80.5
GMSFF&SMIC [61]	HRNet-W32	256 × 192	74.9	80.3
RTMPose [62]	RTMPose-l	256 × 192	73.6	–
Liu et al. [50]	HRNet-W32	256 × 192	75.1	80.2
Ours(PoseNet++)	SHN	256 × 192	**75.4**	**80.8**
HRNet [37]	HRNet-W32	384 × 288	75.8	81.0
HR-ARNet [47]	HRNet-W32	384 × 288	75.9	**90.9**
FEM&MSFF [57]	ResNet-101	384 × 288	74.5	–
HPnet [59]	ResNet-152	384 × 288	75.6	–
GLCFBNet [48]	SHN	384 × 288	76.4	81.5
Liu et al. [50]	HRNet-W32	384 × 288	76.3	81.0
Ours(PoseNet++)	SHN	384 × 288	**76.5**	81.6

**Table 7:** Comparisons on the COCO test-dev2017 set.

Method	Backbone	Input Size	AP	AR
HRNet [37]	HRNet-W32	256 × 192	73.5	78.9
OKS-net [62]	HRNet-W32	256 × 192	73.9	79.3
TokenPose [40]	TokenPose-B	256 × 192	74.0	79.1
HR-ARNet [47]	HRNet-W32	256 × 192	73.9	79.3
EMpose [46]	HRNet-W32	256 × 192	73.8	79.1
FEM&MSFF [57]	ResNet-50	256 × 192	71.6	–
MSPENet [58]	ResNet-50	256 × 192	72.2	77.8
DLIFP [60]	HRNet-W32	256 × 192	73.8	–
GLCFBNet [48]	SHN	256 × 192	74.6	79.8
Liu et al. [50]	HRNet-W32	256 × 192	74.2	79.3
Ours(PoseNet++)	SHN	256 × 192	**74.7**	**79.9**
CPN [25]	ResNet	384 × 288	72.1	78.5
CPN(ensemble) [25]	ResNet	384 × 288	73.0	79.0
HRNet [37]	HRNet-W32	384 × 288	74.9	80.1
OKS-net [63]	HRNet-W32	384 × 288	75.2	80.4
FEM&MSFF [57]	ResNet-50	384 × 288	73.3	–
GLCFBNet [48]	SHN	384 × 288	75.3	80.7
Liu et al. [50]	HRNet-W32	384 × 288	75.4	80.3
Ours(PoseNet++)	SHN	384 × 288	**75.5**	**80.6**

#### 4.5.4. Results on the CrowdPose dataset.

As shown in Table 8, this study compares and analyzes the performance of PoseNet++ with that of the top-performing pose estimation algorithms in recent years on the CrowdPose test set. The experimental results show that PoseNet++ achieves 68.6 and 70.6 APs at input resolutions of 256 × 256 and 384 × 288, which are 0.1 and 0.2 higher compared to the latest (2024) and highest AP method proposed by Liu et al. Compared to SimCC, which is the transformer-based approach, the AP of PoseNet++ is higher by 1.9.

**Table 8:** Comparisons on the CrowdPose test set.

Method	Backbone	Input Size	AP
Mask R-CNN [64]	ResNet	256 × 192	57.2
AlphaPose [65]	AlphaPose	256 × 192	61.0
HRNet [37]	HRNet-W32	256 × 192	67.5
SimCC [43]	HRNet-W32	256 × 192	66.7
Liu et al. [50]	HRNet-W32	256 × 192	68.5
Liu et al. [50]	HRNet-W32	384 × 288	70.4
PoseNet++(Ours)	SHN	256 × 192	**68.6**
PoseNet++(Ours)	SHN	384 × 288	**70.6**

#### 4.5.5. Comparison experiment of model complexity.

**Fig 12:**
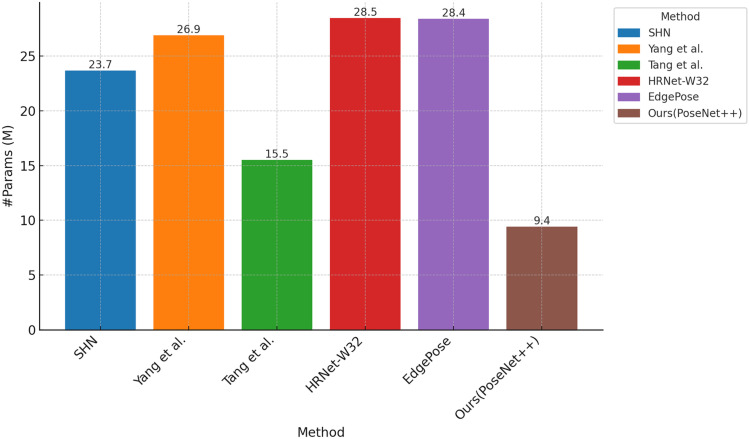
Comparison of model parameter counts using different methods on the MPII dataset.

**Fig 13:**
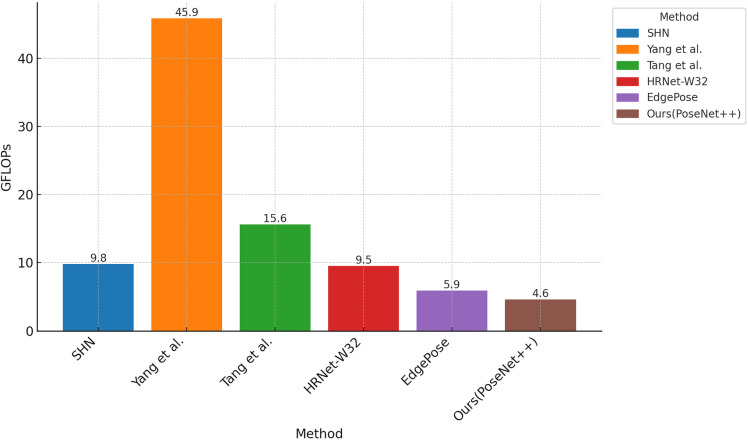
Comparison of GFLOPs using different methods on the MPII dataset.

**Fig 14:**
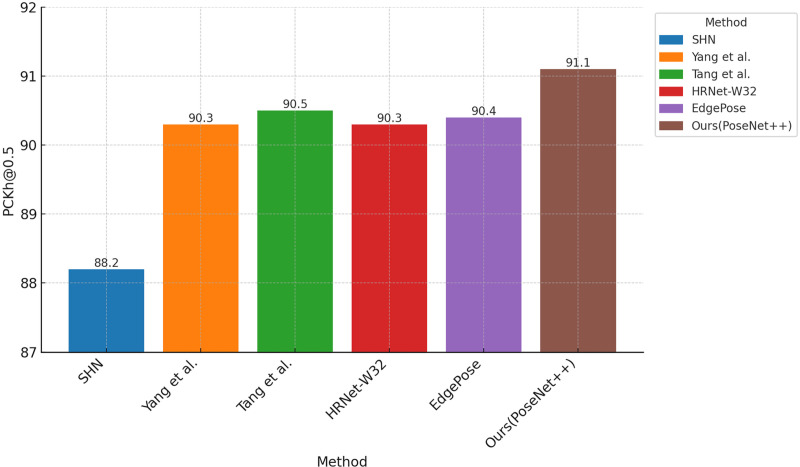
Comparison of PCKh@0.5 using different methods on the MPII dataset.

This section discusses the relationship between #Params and GFLOPs for PoseNet++ and other methods with similar accuracy on the MPII dataset. The comparison results are illustrated in Table 9, Fig 12,13, and Fig 14. As shown in Table 9, PoseNet++ has 9.4M parameters and 4.6 GFLOPs. Compared with the original network [4], the number of model parameters is reduced by 60.3% and the number of floating-point operations is reduced by 53.1%. At the same time, compared to methods with similar accuracy [33,34,37,44], the model parameter count and computational complexity of PoseNet++ are much lower. It is worth mentioning that compared to the latest (2024) EdgePose, PoseNet++ improves the PCKh score by 0.7 while reducing the number of parameters by 19.0M and the computational cost by 1.3 GFLOPs.

**Table 9:** Comparison of model parameters and computational complexity on the MPII validation set (PCKh@0.5).

Method	#Params(M)	GFLOPs	Mean
SHN [4]	23.7	9.8	88.2
Yang et al. [33]	26.9	45.9	90.3
Tang et al. [34]	15.5	15.6	90.5
HRNet-W32 [37]	28.5	9.5	90.3
EdgePose [44]	28.4	5.9	90.4
Ours(PoseNet++)	**9.4**	**4.6**	**91.1**

#### 4.5.6. Comparison experiment of model inference speed.

**Fig 15:**
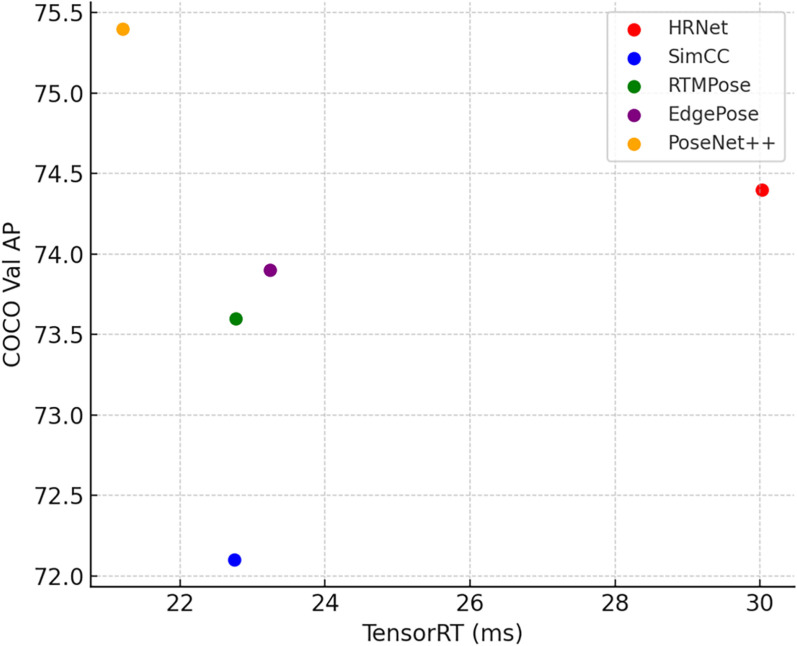
PoseNet++ inference speed in Jetson Xavier NX.

**Fig 16:**
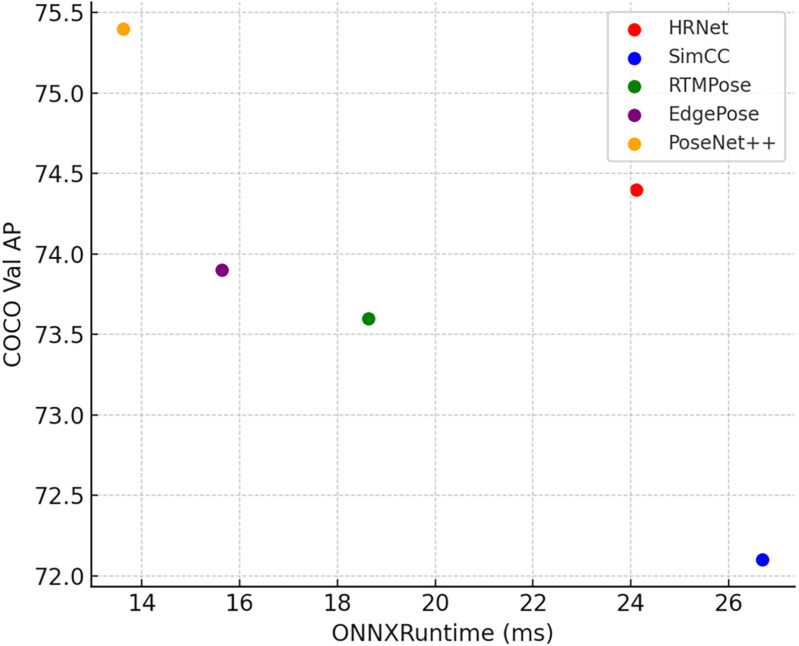
PoseNet++ inference speed in i9-10920X.

We conducted inference and performance testing of PoseNet++ and several commonly used models on the NVIDIA Jetson Xavier NX, leveraging TensorRT for acceleration. Data was exported in single-precision floating-point format (FP32) with a batch size of 1. Speed testing included 50 pre-trains and 1000 inference runs to obtain accurate data. A total of 1000 test images—randomly drawn from the COCO2017 dataset and encompassing both single-person and multi-person scenes—were used to replicate practical deployment conditions. Inference speed was also evaluated on a CPU device (i9-10920X) using the ONNXRuntime format. Experimental findings are illustrated in Table 10, Fig 15, and Fig 16.

**Table 10:** Inference speeds of different methods.

Method	Backbone	Input size	COCO Val AP	TensorRT(ms)	ONNXRuntime(ms)
HRNet [37]	HRNet-W32	256 × 192	74.4	30.03	24.11
SimCC [43]	MobileNet-V2	256 × 192	72.1	22.75	26.69
RTMPose [62]	RTMPose-l	256 × 192	73.6	22.77	18.63
EdgePose [44]	EdgeNet-l	256 × 192	73.9	23.24	15.63
PoseNet++	SHN	256 × 192	**75.4**	**21.21**	**13.62**

On the NVIDIA Jetson Xavier NX device, PoseNet++ exhibited notable performance. When compared to MobileNetV2 using the SimCC method, PoseNet++ delivered a 3.3 AP score improvement while maintaining similar inference speed. PoseNet++ attained an inference speed of 47.1 FPS with a score of 75.4 AP. PoseNet++ surpassed RTMPose-l in performance and exceeded EdgePose by approximately 4.1 FPS. In comparison with HRNet-W32, PoseNet++ improved the AP score by 1.0 while delivering a 13.8 FPS speed gain. On the CPU device (i9-10920X), PoseNet++ showed more impressive speed performance. PoseNet++ offered faster inference speed than both HRNet-W32 and MobileNetV2 using the SimCC method, while also yielding better accuracy. Compared to EdgePose-l, PoseNet++ ran faster and achieved a 1.5 AP score improvement. PoseNet++ achieved a peak of 73.4 FPS and a score of 75.4 AP on CPU, outperforming RTMPose-l by around 19.7 FPS. Overall, PoseNet++ is capable of delivering real-time and accurate human pose estimation even in resource-constrained environments, including on edge devices such as the NVIDIA Jetson Xavier NX.

### 4.6. Qualitative comparisons

In this section, a qualitative comparative analysis between PoseNet++ and the original SHN is presented. While SHN demonstrates strong benchmark performance in human pose estimation, it has difficulty producing reasonable pose estimation results in cases of occlusion, complex scenarios, and challenging poses. SHN suffers from limitations due to the fixed-size RFs of the standard convolutional operations used in its layers and its insufficient ability to model multi-scale features effectively. SHN has difficulty in capturing the larger range of contextual dependencies required to understand the global spatial arrangement of body parts. The network’s ability to handle long-range dependencies between distant joints—such as the relationship between the head, torso, and limbs— is severely limited. This makes it challenging for SHN to reason accurately about joint configurations in situations where body parts are missing, occluded, or exhibit complex deformations. SHN lacks an explicit mechanism to focus on relevant spatial regions. Therefore, the joints of the target person are easily confused with other objects or individuals in complex scenarios involving multiple people or clutter. PoseNet++ addresses these challenges by introducing several key innovations—MSPAHM, C-CPCA, and PBRM. In the design of MSPAHM, 3 × 3 convolutions in the hourglass modules are replaced by MSPA modules. MSPAHM models global dependencies between different joints through feature tensor channel interactions, enabling the network to capture the global context necessary for understanding the spatial arrangement of body parts, even when occlusions or overlapping poses are present. The incorporation of C-CPCA helps retain keypoint positional information and guides the network to focus on critical regions. This mechanism reduces confusion between the target person’s keypoints and background clutter or other individuals in the scene, achieving more accurate localization in complex scenarios. To overcome SHN’s inability to effectively handle multi-scale and complex pose variations, PoseNet++ incorporates PBRM. Compared to the bottleneck residual module, standard 3 × 3 convolution is replaced with PConv, significantly expanding the RF. This enables PoseNet++ to more effectively model the hierarchical structure of the human body and enhances its ability to capture long-range dependencies between body parts. SKConv is incorporated after the second 1 × 1 convolution layer to dynamically select the optimal kernel size based on the input features. This dynamic kernel selection is particularly effective for adaptive multi-scale feature extraction, further improving the network’s capacity to process complex poses and scale variations. Due to the design of MSPAHM and PBRM, which replace the original 3 × 3 convolutions with lightweight modules, the model complexity is significantly reduced. This also reduces the hardware requirements for deploying PoseNet++ in practice, which is crucial for its real-world application on edge devices.

## 5. Discussion

Recent developments in HPE, especially in multi-person scenarios, have opened new opportunities for intelligent surveillance and security-critical applications. Accurate localization of human keypoints not only enables fine-grained behavior analysis but also facilitates abnormal behavior detection in complex environments such as smart buildings, airports, and industrial facilities. The VREMD framework [66] focuses on specific areas of the human body and joints through Transformer-based attention mechanism and motion disentanglement techniques. Therefore, this design can improve pose estimation performance. These motion-aware representations can effectively capture abnormal behaviors such as loitering, climbing, or abrupt changes in direction activities often flagged in surveillance contexts. PoseNet++, as presented in this study, achieves high accuracy and low complexity under multiple challenging conditions. Its efficient and compact design renders it highly applicable for real-time deployment in environments with limited computational resources, a common requirement in edge-based surveillance systems.

The value of HPE can also be extended to intrusion detection systems (IDS). While traditionally designed to detect unauthorized access or cyberattacks in digital domains, IDS are increasingly incorporating computer vision components to achieve more comprehensive situational awareness. Recent research in hybrid AI-driven IDS has shown how deep learning models are capable of extracting and classifying temporal patterns indicative of malicious behavior. One study [67] employed an integrated framework leveraging Convolutional Neural Networks (CNNs) and Long Short-Term Memory (LSTM) units, enhanced by Particle Swarm Optimization (PSO) and Grey Wolf Optimization (GWO), to improve detection of False Data Injection Attacks (FDIAs) in smart grid systems. Another study [68] systematically compared conventional algorithms in machine learning, including methods like K-Nearest Neighbors (KNN), Decision Trees, and Naive Bayes, for detecting cyberattacks, demonstrating that feature selection and ensemble methods significantly enhance IDS performance. Although these methods primarily focus on digital threat detection, they exemplify how AI-based systems can be effectively designed to process high-dimensional, sequential data—paralleling the temporal structure of pose-based behavior sequences in surveillance footage. This suggests that pose estimation could serve as a visual perception module within hybrid IDS frameworks, enabling real-time detection of both physical intrusions and behavioral anomalies. Such interdisciplinary integration would expand the applicability and impact of pose estimation technologies. Particularly, PoseNet++ offers structured representations of human activities, contributing to improved scene understanding and automated decision-making. Its lightweight architecture makes it well-suited for deployment on edge computing platforms, further supporting its real-world use in scalable, distributed monitoring systems.

## 6. Conclusion and future scope

This paper proposes PoseNet++. It is an innovative and efficient deep learning architecture tailored for high-precision human pose estimation. We carried out comprehensive experiments on four public datasets—MPII, LSP, COCO2017, and CrowdPose. The results confirmed that PoseNet++ delivers state-of-the-art accuracy while significantly reducing model complexity. The integration of MSPAHM, C-CPCA, and PBRM enhances keypoint localization across diverse and challenging conditions, offering improved computational efficiency suitable for real-time applications.

In robotics, PoseNet++’s accurate human pose estimation is critical for safe human-robot interaction (HRI), collaborative manufacturing, and gesture-based robot control. PoseNet++’s low-latency and lightweight design also makes it highly adaptable to embedded robotic platforms and autonomous systems. In biomedicine, our framework holds promise for applications such as physical rehabilitation monitoring, movement disorder diagnosis, and ergonomic assessments, where fine-grained analysis of body movement is essential. The high spatial precision of PoseNet++ could also facilitate non-intrusive patient monitoring and real-time biofeedback systems. In the realm of augmented and virtual reality (AR/VR), PoseNet++ can be leveraged to enable full-body tracking, immersive avatar control, and enhanced spatial awareness, all of which are fundamental to next-generation interactive environments. Its lightweight structure is especially conducive to edge computing devices such as AR headsets and mobile platforms. Future work will investigate domain adaptation strategies to extend PoseNet++ to varied environments and diverse populations, as well as its integration with multimodal perception systems for holistic understanding of human behavior.
